# Malaria and Dengue Co-infection: A Comprehensive Study in Peshawar, Pakistan

**DOI:** 10.7759/cureus.76401

**Published:** 2024-12-26

**Authors:** Mehwash Iftikhar, Mian Mufarih Shah, Wazir Muhammad Khan, Zia Uddin, Muhammad Numan Saleem, Sheraz J Khan

**Affiliations:** 1 Internal Medicine, Medical Teaching Institution (MTI) Hayatabad Medical Complex, Peshawar, PAK; 2 Internal Medicine, Medical Teaching Institution (MTI) Khyber Teaching Hospital, Peshawar, PAK; 3 Internal Medicine, Medical Teaching Institution (MTI) Lady Reading Hospital, Peshawar, PAK

**Keywords:** co-infection, dengue, epidemiology, malaria, pakistan, urban health

## Abstract

Background: Malaria and dengue are significant mosquito-borne diseases prevalent in tropical and subtropical climates, with increasing reports of co-infections. This study aimed to determine the frequency, patterns, and risk factors of these co-infections in Peshawar.

Methods: A cross-sectional study was conducted from June to December 2023 in three tertiary care hospitals in Peshawar. We evaluated 322 febrile patients (temperature >38 °C) using dengue serological tests (NS1, IgM, IgG), malarial parasite microscopy (thick and thin films with double-reading quality control), and immunochromatography. Clinical and demographic data were collected using structured questionnaires. Statistical analysis was performed using SPSS Version 26 (IBM Corp., Armonk, NY).

Results: Of 322 patients (mean age 35.7 ± 14.2 years; 187 male, 58.1%), 72 (22.4%) had malaria-dengue co-infection. Co-infected patients showed NS1 positivity in 30/72 (41.7%), with higher rates in urban areas (60/231 [26.0%] vs. 12/91 [13.2%]; odds ratio [OR] = 2.31, 95% confidence interval [CI]: 1.18-4.52, *P* < 0.01). Age (*r *= 0.15, *P *= 0.007) and education (OR = 1.84, 95% CI: 1.09-3.12, *P *= 0.02) were significant risk factors. Co-infected patients had longer hospital stays (5.8 ± 2.3 vs. 3.9 ± 1.8 days, *P *< 0.001) and higher mortality (2.8% vs. 1.2%, *P *= 0.04).

Conclusions: Co-infection of malaria and dengue is common in Peshawar, particularly in urban areas and during monsoon seasons. Age and educational status are significant risk factors. These findings necessitate routine screening for both diseases in febrile patients from endemic areas, especially during peak seasons. Targeted surveillance and vector control strategies in urban areas are recommended.

## Introduction

Malaria and dengue represent two of the most significant vector-borne diseases affecting global health. The World Health Organization has identified these diseases as major public health concerns, with annual global malaria cases exceeding 240 million and dengue cases surpassing 100 million, particularly alarming in regions where their endemic zones overlap [1]. Recent surveillance data indicates a worrying expansion of affected geographical areas, complicated by climate change and rapid urbanization, with co-infections emerging as a significant challenge. Understanding these co-infections has become increasingly crucial, as comprehensive analyses demonstrate their growing significance in tropical and subtropical healthcare systems, where limited resources often face dual epidemiological challenges [2,3].

The historical context of these diseases presents a striking contrast in human disease evolution. Malaria's presence in human populations dates back millennia, as evidenced in paleopathological studies that traced its impact through ancient civilizations and its role in shaping human genetic adaptations, including the evolution of hemoglobinopathies and other protective genetic traits [4]. In contrast, dengue emerged as a global health concern more recently, with documentation of the first major outbreaks in Southeast Asia during the 1950s, marking the beginning of its worldwide spread through increased global travel, urbanization, and changes in vector ecology [5,6].

The dynamics of co-infection present unique challenges due to distinct vector characteristics and transmission patterns. Pioneering work in understanding co-infection patterns revealed that varying incubation periods - 3-14 days for dengue and 7-30 days for malaria - can lead to complex clinical presentations that challenge conventional diagnostic protocols [7,8]. This ecological overlap has profound implications for disease surveillance and control strategies. Global analysis of disease distribution patterns reveals how modern environmental conditions in endemic regions increasingly support both Anopheles and Aedes aegypti populations, creating unprecedented opportunities for concurrent transmission [9,10].

Pakistan presents a compelling case study of these global challenges, particularly in rapidly urbanizing regions. Both diseases maintain endemic status throughout the country, with significant seasonal variations affecting their prevalence [11,12]. Historical data shows a gradual increase in co-infection rates, particularly in urban areas, with reported rates varying from 4.3% to 23.2% across different Pakistani cities [13,14,15]. These patterns are influenced by monsoon seasons and changing agricultural practices but are increasingly affected by urban development and climate change [16].

The vector biology underlying these co-infections presents complex ecological interactions. Research has documented the historical spread and breeding patterns of disease vectors [17,18], while subsequent studies have revealed their adaptations to urban environments [19,20]. These environmental modifications create unique microenvironments supporting both vectors, with temperature variations significantly affecting transmission efficiency [21,22].

The immunological interface between these pathogens presents unique challenges in co-infected individuals. Studies have demonstrated significant cross-reactivity between malaria antigens and dengue antibodies [23,24], potentially modifying disease progression and severity. This interaction is particularly relevant in endemic regions where repeated exposure to both pathogens may shape immune responses [25,26]. Recent systematic reviews and meta-analyses have shown distinct patterns in disease manifestation and severity [27,28,29,30], with important implications for clinical management.

## Materials and methods

Study design and setting 

This cross-sectional study was conducted from June to December 2023 at three tertiary care hospitals in Peshawar, Pakistan: Hayatabad Medical Complex, Khyber Teaching Hospital, and Lady Reading Hospital. The study protocol was approved by the Ethical Review Board of Hayatabad Medical Complex (approval number: HMC/ERC/2023/156), and written informed consent was obtained from all participants or their guardians.

Sample size calculation 

The sample size was calculated using the formula *n* = *Z*²*P*(1 - *P*)/*d*², where *Z* = 1.96 (95% confidence interval [CI]), *P* = 23.2% (based on previous regional studies of co-infection prevalence), and *d* = 5% precision level, with a power of 80%. The calculated minimum sample size was 275 patients. To account for potential dropouts and incomplete data, we increased this by 15%, yielding a final target of 316 participants. Ultimately, 322 patients were enrolled through consecutive sampling.

Participants selection 

The study included patients aged 12 years or older, presenting with fever (temperature >38 °C) lasting less than 14 days, who provided informed consent. We excluded pregnant women and patients with severe comorbidities, including end-stage renal disease (eGFR < 15 mL/minute/1.73 m²), chronic liver disease (Child-Pugh class C), and active malignancies. Patients receiving immunosuppressive therapy or those with incomplete clinical data or inadequate blood samples were also excluded.

Data collection 

A structured, pre-tested questionnaire captured demographic information including age, gender, education level, and residence. Education level was categorized as uneducated (no formal schooling) or educated (no formal education), while residence was classified as urban or rural based on local administrative definitions. Clinical history included fever duration, associated symptoms, and prior treatments, along with environmental factors such as travel history and vector exposure.

Clinical severity was evaluated using a standardized scoring system that assessed vital signs (temperature, blood pressure, and respiratory rate), laboratory parameters (complete blood count, liver function tests, and renal function tests), and organ function indicators (Glasgow Coma Scale, oxygen saturation, and urine output). Treatment requirements, including oxygen support, vasopressor need, and ventilation status, were documented. Daily monitoring encompassed severity score calculation, treatment modifications, complication documentation, and clinical outcome assessment.

Laboratory methods

Blood samples (5 mL) were collected using a standard aseptic technique and processed within two hours of collection. Samples for serology were stored at 2-8 °C, while blood smears were maintained at room temperature until analysis.

For dengue diagnosis, we performed NS1 antigen testing using Enzyme-Linked Immunosorbent Assay (ELISA; Panbio Dengue Early ELISA, Abbott, Abbott Park, IL), which had a sensitivity of 95.8% and a specificity of 98.2%. Dengue-specific IgM and IgG antibody detection utilized Panbio Dengue IgM/IgG capture ELISA (Abbott), with cutoff values determined according to the manufacturer's guidelines. Quality control measures included running positive and negative controls with each batch, external quality assessment every three months, and inter-laboratory comparison with a reference laboratory.

Malaria diagnosis employed both thick and thin blood films stained with 3% Giemsa stain for 45 minutes. Slides were examined under oil immersion (x1,000 magnification) by experienced microscopists who screened a minimum of 200 fields before declaring a slide negative. Species identification and parasite density were determined using standardized protocols. Quality control included double-reading of all positive slides and a 10% random selection of negative slides, with discrepancies resolved by a third senior microscopist. Monthly proficiency testing of microscopists was conducted. Additionally, rapid immunochromatographic testing for Plasmodium falciparum and Plasmodium vivax (SD BIOLINE Malaria Ag P.f/P.v) was performed according to the manufacturer's instructions, with internal quality control for each test batch.

Statistical analysis 

Data analysis employed SPSS version 26.0 (IBM Corp., Armonk, NY), with double entry and validation procedures. Data normality was assessed using the Kolmogorov-Smirnov test. Missing data, which constituted less than 5% for any variable, were handled using complete case analysis. Continuous variables were expressed as mean ± standard deviation or median (interquartile range) based on distribution, while categorical variables were presented as frequencies and percentages. Ninety-five percent CIs were calculated for primary outcomes.

Between-group comparisons utilized independent t-tests or Mann-Whitney U tests for continuous variables based on distribution normality. Categorical variables were analyzed using chi-square tests or Fisher's exact test when expected frequencies were less than 5. Multivariate logistic regression identified co-infection predictors, with initial variable selection based on univariate *P*-values less than 0.2, followed by stepwise backward elimination. Final model retention required *P*-values less than 0.05. Model diagnostics included the Hosmer-Lemeshow goodness-of-fit test, the area under the Receiver Operating Characteristic (ROC) curve analysis, and variance inflation factor assessment for collinearity.

Correlation analysis employed Pearson's or Spearman's correlation coefficients for normally distributed and non-normal/ordinal variables, respectively. All statistical tests were two-sided, with significance set at *P *< 0.05. Test statistics (t-value for t-tests, χ² for chi-square tests, and U for Mann-Whitney tests) were calculated and reported alongside *P*-values for all comparisons.

## Results

Among the 322 enrolled patients, 72 (22.4%) were diagnosed with malaria-dengue co-infection. The mean age of participants was 35.7 ± 14.2 years, with males comprising 187/322 (58.1%) of the sample. Urban residents constituted 231/322 (71.7%) of the study population, while 198/322 (61.5%) were categorized as uneducated (Table 1). 

**Table 1:** Demographic and clinical characteristics of the study population (N = 322). ^§^*P* < 0.05 considered significant. ^*^Values presented as mean ± SD. ^†^Clinical features presented as *n* (%). ^‡^Median (IQR). SD, standard deviation; IQR, interquartile range U: Mann-Whitney U test

Characteristic	Total population (*N *= 322)	Co-infected (*n *= 72)	Non-co-infected (*n *= 250)	Test statistic	*P*-value^§^
Age (years)*	35.7 ± 14.2	38.2 ± 13.7	34.9 ± 14.3	*t* = 2.18	0.03
Gender				*χ*² = 0.57	0.45
Male	187 (58.1)	44 (23.5)	143 (76.5)		
Female	135 (41.9)	28 (20.7)	107 (79.3)		
Education				*χ*² = 5.38	0.02
Uneducated	198 (61.5)	36 (18.2)	162 (81.8)		
Educated	124 (38.5)	36 (29.0)	88 (71.0)		
Residence				*χ*² = 6.42	<0.01
Urban	231 (71.7)	60 (26.0)	171 (74.0)		
Rural	91 (28.3)	12 (13.2)	79 (86.8)		
Clinical Features^†^					
Fever duration^‡^	5 (3-7)	6 (4-8)	5 (3-7)	*U* = 7624	0.04
Headache	245 (76.1)	58 (80.6)	187 (74.8)	*χ*² = 1.03	0.31
Myalgia	198 (61.5)	47 (65.3)	151 (60.4)	*χ*² = 0.57	0.45
Joint pain	167 (51.9)	39 (54.2)	128 (51.2)	*χ*² = 0.19	0.66
Vomiting	143 (44.4)	35 (48.6)	108 (43.2)	*χ*² = 0.65	0.42

The clinical presentation varied between co-infected and non-co-infected groups, with co-infected patients showing longer fever duration (median 6 days vs. 5 days, *P *= 0.04). Other symptoms, including headache (245/322, 76.1%), myalgia (198/322, 61.5%), joint pain (167/322, 51.9%), and vomiting (143/322, 44.4%), showed no significant differences between groups.

Analysis of dengue serological patterns among co-infected patients revealed a predominance of acute infection. NS1 antigen positivity was detected in 30/72 (41.7%) patients, while IgM positivity and IgG positivity were found in 24/72 (32.8%) and 18/72 (25.5%) patients, respectively. In terms of malarial species, P. vivax predominated in 49/72 (68.0%) cases, while P. falciparum was found in 23/72 (32.0%) cases, with no mixed infections observed (Table 2). 

**Table 2:** Distribution of dengue serological patterns and malarial species in co-infected patients (n = 72). Values presented as *n* (%). - indicates not applicable. CI, confidence interval

Parameter	Number (%)	95% CI
Dengue serological pattern		
NS1 positive (Acute)	30 (41.7)	30.2-53.9
IgM positive (Recent)	24 (32.8)	22.3-44.9
IgG positive (Past)	18 (25.5)	16.2-37.1
Malarial species		
Plasmodium vivax	49 (68.0)	56.2-78.3
Plasmodium falciparum	23 (32.0)	21.7-43.8
Mixed infection	0 (0.0)	-

Co-infected patients demonstrated significantly higher severity scores and longer hospital stays compared to those with single infections. The significantly higher rates of complications in co-infected patients were accompanied by increased mortality. Notably, the odds ratio (OR) for intensive care admission in co-infected patients was 2.41 (95% CI: 1.14-5.08, *P *< 0.01), indicating substantially higher resource utilization in this group. A comprehensive comparison of clinical outcomes between co-infected and single-infection patients is presented in Table 3.

**Table 3:** Comparison of clinical outcomes between co-infected and single-infection patients. ^*^Values presented as mean ± SD. ^§^Values presented as *n* (%). ^†^*t* = Student's t-test; *χ*² = Chi-square test. ^‡^*P *< 0.05 considered significant. SD, standard deviation

Parameter	Co-infected (*n* = 72)	Single infection (*n *= 250)	Statistical test^†^	*P*-value^‡^
Clinical severity score*	8.4 ± 2.1	6.2 ± 1.8	*t* = 8.42	<0.001
Hospital stay (days)*	5.8 ± 2.3	3.9 ± 1.8	*t* = 7.16	<0.001
Intensive care admission^§^	13 (18.1)	21 (8.4)	*χ*² = 5.82	<0.01
Severe thrombocytopenia (<50,000/µL)	22 (30.6)	45 (18.0)	*χ*² = 5.24	<0.01
Acute kidney injury	15 (20.8)	28 (11.2)	*χ*² = 4.52	0.03
Severe hepatitis	12 (16.7)	22 (8.8)	*χ*² = 3.86	0.04
Case fatality^§^	2 (2.8)	3 (1.2)	Fisher's exact	0.04

Multivariate analysis identified several significant predictors of co-infection as shown in Table 4. Age demonstrated a positive correlation with co-infection risk (*r* = 0.15, *P* = 0.007). Urban residence and educational status emerged as significant independent predictors of co-infection, while gender and occupation showed no significant association (Table 4). 

**Table 4:** Multivariate analysis of factors associated with malaria-dengue co-infection. ^*^Correlation coefficient (*r*) = 0.15. ^†^Occupation categories based on primary employment status. - indicates not applicable. *P *< 0.05 considered significant. OR, odds ratio; CI, confidence interval

Variable	Univariate OR (95% CI)	Multivariate OR (95% CI)	Wald *χ*²	*P*-value
Age (per year)*	1.02 (1.00-1.04)	1.03 (1.01-1.05)	7.24	0.007
Urban residence	2.31 (1.18-4.52)	2.45 (1.23-4.88)	6.42	<0.01
Educational status	1.84 (1.09-3.12)	1.92 (1.11-3.32)	5.38	0.02
Gender (Male)	1.17 (0.78-1.76)	1.14 (0.74-1.75)	0.57	0.45
Occupation^†^				
Indoor work	1.0 (Reference)	1.0 (Reference)	-	-
Outdoor work	1.45 (0.88-2.39)	1.38 (0.82-2.32)	1.44	0.23
Student	1.22 (0.69-2.15)	1.19 (0.66-2.14)	0.34	0.56

Temporal analysis revealed distinct seasonal patterns in co-infection rates as presented in Table 5. Peak transmission occurred during the monsoon season (August-September), with 43/72 (59.7%) cases documented during this period. The urban-to-rural ratio showed marked seasonal variation, reaching 5:1 during peak season compared to 3:1 during other months. Environmental parameters demonstrated strong correlations with case numbers, particularly rainfall (*r* = 0.78) and relative humidity (*r* = 0.65) (Table 5).

**Table 5:** Seasonal distribution and urban-rural patterns of co-infection cases. ^*^Environmental data presented as mean ± standard deviation (SD). - indicates not applicable. *P *< 0.05 considered significant.

Parameter	Peak season (Aug-Sep)	Other months	Total	Test statistic	*P*-value
Number of cases, *n* (%)	43 (59.7)	29 (40.3)	72 (100)	*χ*² = 8.42	<0.01
Urban-rural distribution					
Urban cases	36 (83.7)	24 (82.8)	60 (83.3)	*χ*² = 0.01	0.91
Rural cases	7 (16.3)	5 (17.2)	12 (16.7)		
Urban:rural ratio	5:1	3:1	5:1		
Environmental factors					
Average rainfall (mm)	189.5 ± 45.2	42.3 ± 12.8	-	*t* = 18.24	<0.001
Mean temperature (°C)	29.8 ± 2.4	24.5 ± 3.2	-	*t* = 8.76	<0.001
Relative humidity (%)	75.6 ± 5.8	62.4 ± 6.9	-	*t* = 9.12	<0.001

## Discussion

Our study reveals a substantial malaria-dengue co-infection rate of 72/322 (22.4%) in Peshawar, aligning with Assir et al.'s comprehensive study in Lahore reporting 23.2% co-infection rates [11], though standing significantly higher than the 4.3% reported by Saqib et al. in Rawalpindi [12]. Such regional variations, as documented by Kraemer et al., likely reflect complex interactions between urbanization, vector ecology, and human population dynamics [13]. These patterns are further supported by Nikolich-Žugich's extensive review of vector-borne diseases in developing regions [14] and Halstead's observations regarding urban vulnerability to vector-borne diseases [15].

The serological patterns observed in our study, particularly the predominance of acute dengue (30/72, 41.7%) among co-infected cases, provide crucial insights into the temporal dynamics of co-infection. These findings align with the infection sequence patterns documented by Epelboin et al. [16] and Mohapatra et al. [17]. The species distribution in our cohort (49/72, 68% P. vivax; 23/72, 32% P. falciparum) differs notably from patterns observed in neighboring regions by Faruque et al. [18], suggesting local ecological influences on species prevalence, a phenomenon further supported by Khan et al.'s regional analyses [19] and Epelboin et al.'s comparative studies [20].

The observed urban predominance in our study, with an urban-to-rural ratio reaching 5:1 during peak seasons, reflects patterns documented in systematic reviews by Powell and Tabachnick [21] and Shragai et al. [22]. Studies of vector breeding patterns by Ryan et al. [23] and urban environmental analyses by Souza-Neto et al. [24] provide mechanistic explanations for these urban-rural disparities. The influence of urban infrastructure on vector distribution has been well-documented by Minakawa et al. [25], particularly regarding the creation of unique microenvironments supporting both vectors.

The seasonal clustering of cases in our study, with 43/72 (59.7%) occurring during the monsoon season, demonstrates clear temporal patterns. The strong correlation between case numbers and environmental parameters (rainfall: *r* = 0.78, relative humidity: *r* = 0.65) aligns with comprehensive analyses by Rodriguez-Morales et al. [26] and Rathore et al. [27]. These environmental associations are further supported by systematic reviews of climate-driven transmission dynamics by Kumar et al. [28] and Shepard et al. [29].

Our finding of increased co-infection risk with age (OR = 1.03, 95% CI: 1.01-1.05) merits particular attention. This association likely reflects cumulative exposure risk and age-related changes in immune response, as documented in systematic reviews by Kotepui and Kotepui [30]. The positive correlation between education level and co-infection risk (OR = 1.92, 95% CI: 1.11-3.32) challenges traditional socioeconomic health gradients but may reflect occupational and behavioral patterns associated with urban living, as emphasized in analyses by Waggoner et al. [31] and current WHO guidelines [32].

Our study's strengths include its comprehensive diagnostic approach, robust sample size, and multi-center design. The inclusion of both microscopic and serological testing enhances diagnostic reliability, while our detailed environmental analysis provides valuable context for transmission patterns. However, several limitations warrant consideration. First, our single-season sampling may not capture annual variations in co-infection patterns. Second, the exclusion of patients under 12 years limits generalizability across age groups. Third, while we documented clinical presentations, we did not assess long-term outcomes or complications. Finally, our urban hospital-based sampling may have introduced selection bias, potentially overestimating urban prevalence.

Moving forward, these findings highlight crucial implications for public health intervention in endemic regions, as outlined in recent systematic reviews [33,34]. Healthcare systems should implement enhanced surveillance and screening protocols, particularly in urban areas during peak seasons. Priority should be given to strengthening vector control measures, developing rapid diagnostic algorithms, and establishing climate-informed early warning systems. Implementation of these evidence-based strategies could significantly reduce disease burden and improve health outcomes in endemic regions worldwide. 

## Conclusions

These findings underscore critical public health implications for endemic regions, necessitating enhanced healthcare surveillance, screening protocols, and vector control measures, particularly in urban areas during peak seasons, alongside the development of rapid diagnostic algorithms and climate-informed early warning systems. For urban zones, a comprehensive surveillance approach incorporating vector density surveys and real-time case reporting is essential, while rural areas require mobile health units, community health worker training, and telemedicine support integrated with existing primary healthcare infrastructure. The implementation strategy should emphasize targeted educational programs, longitudinal research for understanding co-infections, and a three-phase resource allocation model spanning 24 months that accounts for population density, seasonal variations, and emergency response needs, ultimately aiming to reduce disease burden and improve health outcomes in endemic regions worldwide through evidence-based interventions.​​
